# Models for Integrated HIV-Hypertension Care: Comparative Analysis of Six Integration Approaches from Implementation Research Projects Across Africa

**DOI:** 10.21203/rs.3.rs-9464167/v1

**Published:** 2026-06-17

**Authors:** Pooja Gala, Anchindika Mugala, Matthew D Hickey, Claudia E Ordonez, Sarah Gimbel, Karla I Galaviz, Samanta T Lalla-Edward, John Baptist Kiggundu, Daniel Akulebe Henry, Gaone Edwin Mogaetsho, Angela Aifah, Anne Katahoire, Fred Semitala, Lisa R Hirschhorn

**Affiliations:** New York University; Centre for Infectious Disease Research in Zambia; University of California, San Francisco; Emory University; University of Washington; Indiana University Bloomington; University of the Witwatersrand; Makerere University; University of Abuja Teaching Hospital; University of Botswana; New York University; Makerere University; Makerere University; Northwestern University

**Keywords:** HIV, cardiovascular disease, hypertension, integrated care, implementation science

## Abstract

**Introduction:**

In 2020, the National Heart, Lung and Blood Institute formed the Heart, Lung, and Blood Co-morbiditieS IMplementation Models in People Living with HIV (HLB SIMPLe) Alliance, funding implementation research projects in six countries in Africa including Botswana, Mozambique, Nigeria, South Africa, Uganda and Zambia to test diverse models for integrating hypertension services into routine HIV care. We describe key similarities and differences amongst the integration models.

**Methods:**

This sequential explanatory mixed-methods study used online structured questionnaires and meetings to gather quantitative and qualitative data from each project. Integration was defined as the delivery of HIV and hypertension care concurrently for people living with HIV, specifically across the WHO health system building blocks: service delivery (care delivered at the same time/same location), health workforce (same team of healthcare providers), health information systems (merging of information systems), access to essential medicines (medications for both HIV and hypertension in the same pharmacy), and health financing (integrated funding). Data were analyzed using descriptive statistics and rapid deductive content analysis. We then used this data together with the WHO Health System Building Blocks framework to describe HIV-hypertension integration models across key aspects of the health system.

**Results:**

All models fully integrated service delivery and health workforce, often using task sharing to address workforce shortages. Full integration of health information systems was observed in 5/6 projects, and integrated access to essential medication in all projects. Integration occurred at the primary healthcare level and excluded complicated hypertension cases. Only 2/6 projects had integrated health financing for HIV/hypertension care.

**Conclusions:**

This examination highlights high convergence in the design of HIV and hypertension care integration models across WHO Health System Building Blocks despite different study settings, with the main difference being divergent mechanisms of health financing for integrated HIV/hypertension care. Lessons learned will help develop a core set of guidelines for building country-specific models for integrating hypertension and HIV care across different African settings.

**Trial Registration:**

NCT05414526 / NCT05002322 / NCT05031819 / NCT05846503 / NCT05609513 / NCT05005130 / NCT05950919

**Clinical Trial Numbers:**

NCT05414526 (1/31/2023–8/31/2025) (Botswana), NCT05002322 (2/13/2023–12/31/2025) (Mozambique), NCT05031819 (10/26/2022–4/30/2025) (Nigeria), NCT05846503 (6/1/2023–5/31/2025) (South Africa), NCT05609513 (02/06/2023–10/3/2025) (Uganda), NCT05005130 (9/20/2021–12/23/2022); NCT05950919 (05/30/2023–12/01/2025) (Zambia)

## Background

HIV continues to be a serious public health issue, with an estimated 39.9 million people living with HIV (PLWH) worldwide, over half (52%) living in Africa [1]. The scale-up of antiretroviral therapy (ART) has been immensely successful, transforming HIV from a life-threatening condition to a manageable chronic disease with life expectancy now comparable to that of the general population [2, 3]. However, due to inflammation and activation of the innate and adaptive immune responses amplified by chronic HIV, PLWH are developing other chronic conditions, such as hypertension and cardiovascular disease (CVD), at rates higher than the general population [4–6]. Addressing hypertension, a modifiable contributor of CVD, is an important step towards reducing excess CVD morbidity and mortality among PLWH [7, 8].

This more common multi-morbidity has led to a push for integrated health care to address gaps in access to effective care [9–11]. Recent WHO guidelines define integrated care as high-quality comprehensive health services delivered by multidisciplinary teams throughout the life course of an individual with the goal of using resources effectively and strengthening people-centered health systems [12, 13]. In practice, researchers in Port-au-Prince, Haiti [14], Uganda [15–18], Tanzania [18], and Kenya [16, 17] integrated hypertension care into existing HIV clinics [14, 15], or created distinct chronic disease clinics [16–18]. In most studies, integration of HIV and CVD care improved hypertension and CVD outcomes, access to HIV services, reduced HIV stigma, and improved ART adherence [9, 10]. WHO guidelines now recommend that HIV care be integrated with hypertension, diabetes, and mental health services, however there is limited guidance on how to achieve this integration and which strategies may be most effective for supporting implementation [12, 19, 20]. With the abrupt disruptions to global HIV funding commitments in early 2025 and concerns about sustainability of vertical single-disease programs, many countries are urgently grappling with the most effective ways to integrate HIV and general primary health systems, including hypertension care.

In 2020, the National Heart, Lung and Blood Institute formed the **H***eart*, **L***ung, and* **B***lood Co-morbiditie***S IM***plementation Models in* **P***eople* **L***iving with HIV* (HLB SIMPLe) Alliance [21] to support implementation research studies in six countries [22–27]. The Alliance’s primary objective was to assess how effective different models of HIV-hypertension integration were across diverse low- and middle-income (LMIC) settings at improving clinical outcomes. Each project independently developed and applied a contextspecific integration model, tailored to the available resources, the HIV service delivery structure and national health system context. HLB-SIMPLe included two phases: 1) UG3: development and pilot testing strategies/model to achieve integrated HIV and hypertension care, and 2) UH3: implement and test UG3 strategies in real-world settings.

The objective of this study is to characterize and compare each country project’s model of integrated HIV and hypertension care developed in the UG3 phase. The WHO Health System Building Blocks framework was chosen to provide a comprehensive organization of key healthcare system components and a basis for comparison [28]. Insights from this study are important to identifying integration models and strategies for HIV and hypertension that can improve patient outcomes and resource allocation in LMICs.

## Methods

### Overview of Study Design and Methods

We designed a sequential explanatory mixed methods study using online structured questionnaires to collect quantitative data from each HLB SIMPLe Alliance project, followed by meetings with co-authors from different projects to gather qualitative data to better understand similarities and differences observed in the quantitative data. Quantitative and qualitative data were synthesized into a cohesive narrative using the WHO Health Systems Building Blocks framework. This study was conducted by the Dissemination and Implementation (D&I) Committee of the HLB SIMPLe Alliance with representatives from each of the six HLB-SIMPLe projects and the HLB-SIMPLe Research Coordinating Center.

### Study Setting

Integration of HIV and hypertension services occurred in the HIV public primary health care setting in Zambia - TASKPEN [26], Uganda – PULESA [22], South Africa – iHEART-SA [23], Mozambique – SCALE-SAIA-HTN [24], Nigeria – MAP-IT [25], and Botswana - InterCARE [27]. Three projects (InterCARE, PULESA, TASKPEN) integrated hypertension services into pre-existing HIV clinics and one (MAP-IT) integrated hypertension services into a general primary care clinic. Two projects were conducted in settings where HIV and hypertension care were already integrated (SCALE-SAIA-HTN, iHEART-SA). These two projects focused on strengthening and sustaining integrated care to ensure high-quality and comprehensive care for PLWH and hypertension [29, 30].

### Study Population

Primary data collection involved questionnaires and meetings with co-authors representing each of the six projects.

### Data Collection

We used the WHO Health System Building Blocks to design the questionnaire and as an analytical framework to describe integrated care across models. This framework is comprised of six core components of a health system, adapted and defined as follows: 1) *Integrated service delivery* as the co-localization of HIV and hypertension care delivery at the same primary healthcare location at the same time. 2) *Integrated health workforce* as HIV and hypertension care administered by the same healthcare provider team (physician [completed medical school and residency], medical officer [MO - completed medical school and an intern year], or advanced practice providers [APP - prescribers under supervision of a physician or MO] and nurses, community health workers (CHWs) and ancillary staff). 3) *Integration of health system financing* as hypertension and HIV care and medications funded by the same entity. 4) Integrated medical products, vaccines, and technology was narrowed to *integrated access to essential medications and/or basic equipment*, as the disbursement of both HIV and hypertension medications at the same pharmacy. Finally, 5) *integration of health information systems* as use of the same medical record to document both HIV and hypertension clinical history, physical exams, and management plans. 6) *Integration within leadership and governance* across the health system was beyond the scope of the projects (Table 1).

Online questionnaires included questions across five WHO Health System Building Blocks about the integrated care model designed by each study (Appendix). Online questionnaires were e-mailed to study Principal Investigators (PIs) in early May 2024. PIs identified the appropriate study team member to fill out each questionnaire. Each questionnaire was identified by country but the individual filling out the questionnaire remained anonymous to those conducting the study.

During an in-person late May 2004 HLB SIMPLe D&I Committee meeting including members from all projects, questionnaire results were reviewed and responses were clarified. Additionally, a list of follow-up questions was created to better understand each integration model based on feedback and queries raised from the meeting (Appendix). Virtual zoom meetings were scheduled with each co-author (representing each individual project team). Two researchers (two physician-scientists PG and AM) led semi-structured discussions on the selected five WHO Health System Building Blocks in usual care and integration intervention models. Meetings were conducted in English from June-August 2024 and were audio-recorded and transcribed to capture each integration model in detail.

#### Data Analysis:

Questionnaire responses were summarized using descriptive statistics. The percentage of projects that integrated services within each WHO building block was reported. Two investigators (PG, AM) reviewed all the transcripts and conducted a rapid deductive content analysis of the meeting transcripts [31]. A template was tested for usability and relevance during a D&I Committee Meeting. Two investigators (PG and AM) divided the transcripts and entered summarized data directly into the template. Both analysts reviewed all recorded interviews and transcripts, discussed summarized data and reached consensus on points of disagreement through frequent discussions. The findings were discussed amongst D&I Committee members and the templates were transferred into tables and figures. Study participants provided feedback on the findings of the qualitative analysis.

Mixed methods data integration was conducted throughout the process of data collection and analysis [32]. During qualitative interviews, responses from the quantitative questionnaire were reviewed with the interviewee to provide direct clarification, feedback, and expansion on the pre-identified WHO Building Block themes. During analysis, similarities and differences between project models were also highlighted and qualitative data was used to further explain and expand on the quantitative data collected. Findings were reviewed with the co-authors to validate the results. We adhered to the GRAMMS checklist to report mixed methods results in this manuscript [33, 34].

## Results

Six questionnaires and seven meetings (two for iHEART-SA) were completed.

### Integration Models

The definition of integration within each WHO Health Systems Building Blocks was consistent across projects (Table 2). Similar strategies to achieve integration were used by many projects to strengthen health workforce including practice facilitation [22, 25, 27], task shifting [25–27], and provider training [22, 23, 27]. PULESA prioritized integrated health system financing and access to medications [22, 35, 36]. Unique to SCALE-SAIA-HTN, integrated service delivery was optimized using an iterative quality improvement strategy that included clinic staff identifying and prioritizing areas of improvement and testing out workflow modifications [24] (Table 1).

### WHO Health System Building Blocks

*MO: Medical Officer, APP: advanced practice provider – prescribers such as nurse practitioners (focused on adults), clinical officers, and medical technicians under the supervision of a medical officer or physician

### Health workforce

Four projects expanded workforce for screening by task sharing to lay workers and CHWs and/or task shifting to nurses for initial management of hypertension (InterCARE, PULESA, MAP-IT, TASKPEN) to achieve integrated care. Before and during the intervention period, referrals to MOs, physicians, or APPs occurred for uncontrolled hypertension in all projects (Appendix).

**Figure 1:**
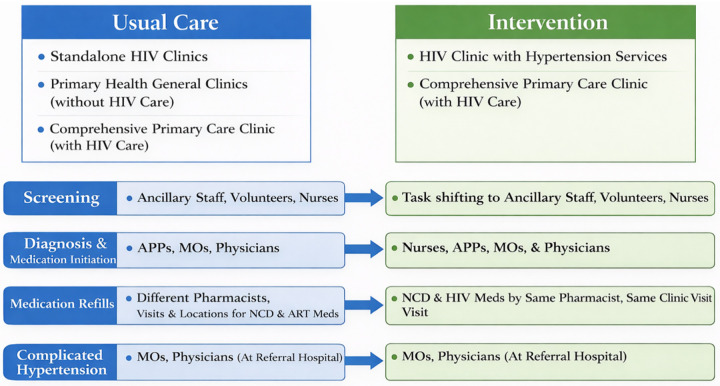
Summary of Integration of Service Delivery and Healthcare Workforce for All Six Projects *MO: Medical Officer, APP: advanced practice provider – prescribers such as nurse practitioners (focused on adults), clinical officers, and medical technicians under the supervision of a medical officer or physician

In qualitative interviews, task shifting emerged as important to strengthen the health workforce and expand access to hypertension care delivery. Existing CHWs and community organizations strengthened screening and linkage to care (MAP-IT). TASKPEN, PULESA, and InterCARE used lay workers trained on the job, CHWs with basic formal training, and volunteers (peers) to perform hypertension screening and support patients in the integrated intervention. In InterCARE and MAP-IT, nurses who often staffed primary care clinics without additional support, were empowered to treat uncomplicated hypertension in the intervention compared to usual care (Fig. 1).

### Service Delivery

In four projects (MAP-IT, PULESA, InterCARE, TASKPEN), prior to the intervention, HIV and hypertension services were provided separately. During the intervention, all projects provided HIV and initial hypertension services (screening, diagnosis, initial treatment, refills for controlled hypertension) in the same clinic visit. For complicated hypertension (patient on more than three medications), or complications due to hypertension, patients were referred to tertiary level care for further management.

In qualitative interviews, integration was described in detail. For MAP-IT, due to staff limitations at primary health care facilities, prior to the intervention, hypertension diagnosis, treatment and refills were only available at secondary care centers. During the intervention, with task shifting these services were provided along with HIV services in primary care settings by nurses (Fig. 1).

### Access to Medications

**Figure 2:**
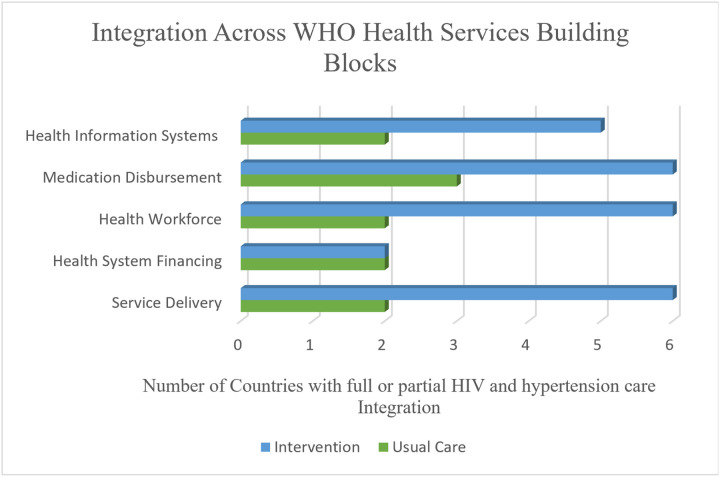
Integration across WHO Health Services Building Blocks

In usual care, there was no consistent model for disbursement of hypertension and HIV medications amongst projects. Only three projects (iHEART-SA, InterCARE, SCALE-SAIA-HTN) disbursed hypertension and HIV medications at the same pharmacy (Fig. 2). Stock outs were common in three projects (SCALE-SAIA-HTN, TASKPEN, PULESA). In integrated care, all projects supported the disbursement of hypertension and HIV medications at the same pharmacy. Stock outs became less frequent except in SCALE-SAIA-HTN.

Qualitative interviews highlighted that patients who could afford to, purchased hypertension medications at external pharmacies when stock outs occurred. Additionally, while HIV medications were free, hypertension medications were out of pocket in usual care for most projects (except InterCARE and iHEART-SA). In integrated care, all projects strengthened medication access by ensuring all medications were available in the same pharmacy at low or no cost.

### Health System Financing

In usual care, HIV and hypertension clinic visits were paid for by the government for five projects. HIV medications and hypertension medications were paid for fully by the government in two projects (InterCARE and iHEART-SA) in usual care and the intervention period. For the remaining projects, medications were paid for by a combination of the government/state, NGOs, pharmaceuticals and patients (Fig. 2). To achieve a model of integrated care, most projects did not change existing health system financing. However, PULESA leveraged an existing pharmaceutical program [35] to provide access to antihypertensives, and when this program closed, continued to procure generic medications at a relatively higher cost. TASKPEN supplemented medicines using a buffer stock when usual supplies ran out. The iHEART-SA study added study-funded staff (nurses and research assistants) to perform initial blood pressure and vitals measurements.

### Health Information Systems

From usual care to integrated care, the use of the same health record system for HIV and hypertension increased from three (2 paper, 1 electronic and paper) to five projects (2 electronic, 2 paper, 1 both electronic and paper) (Fig. 2). Most projects had some form of referral system between primary centers and secondary and tertiary centers, often in the form of letters. This process was not modified by any of the integration interventions.

Qualitative interviews highlighted electricity and internet outages as barriers to consistent usage of an electronic health record (EHR). For SCALE-SAIA-HTN and iHEART-SA, the EHR was used as a tool for data collection and to generate country/district reports to guide quality improvement initiatives. It was not used by clinicians for medical decision making. InterCARE optimized the EHR for clinician documentation and medical decision making. Projects using EHRs increased uptake and optimization of existing systems, but did not build new systems.

## Discussion

We describe six integration models of HIV and hypertension care that are currently being studied in African settings through the NHLBI-funded HLB SIMPLe Alliance [21–27]. Projects used similar strategies to prioritize models that integrated care HIV and hypertension care within the WHO Building Blocks of medication disbursement, health workforce and service delivery. A major challenge to achieving consistent integration of HIV and hypertension care was ensuring a consistent and integrated source of health system financing.

Our findings were consistent with other studies (e.g., INTE-AFRICA, SEARCH) that also defined integrated care through the lens of integrated service delivery (same waiting room, same clinic visit for both hypertension and HIV), health workforce (same prescriber and clinical team managing both hypertension and HIV), and medication disbursement (same pharmacy) [14, 16–18, 37].

Similar to our study, multiple studies identified challenges with integrating health system financing for HIV and hypertension, a critical component to achieving full integration [9, 38–40]. In 2021, almost half of countries in Africa relied on external funding for more than a third of their health expenditures [41]. While HIV has historically received high levels of funding both from domestic and international sources, it was estimated that this funding was 29.3 billion dollars short in 2025 of the funding needed to control HIV/AIDS even before major cuts were made to USAID and PEPFAR [1]. The changing, uncertain funding landscape internationally adds an additional strain in obtaining sufficient funding for consistent access to the medications and equipment needed to achieve full integration of HIV and hypertension care delivery. Medication affordability, particularly, is a major barrier to hypertension treatment persistence and, unless addressed, threatens scale-up and effectiveness of integrated hypertension treatment programs (2). Providing antihypertensives and study staff to take vitals through study funding (e.g., PULESA, iHEART-SA) helped with implementation fidelity. However, for real-world implementation and scale up of the integrated models, countries where medications are not readily accessible or staffing is limited will require more stable, long-term funding sources from the government, NGOs or public-private partnerships to provide medications at a subsidized rate to patients and increase staffing.

Overall, the projects in the HLB SiMPLE Alliance ranged in their generalizability, a natural tension in implementation science trials, often dependent on setting of implementation and local resource availability. Though, as many of the projects in this study have done, leveraging existing HIV infrastructure and resources and choosing practical implementation strategies may be the most feasible way of meeting the funding and infrastructural needs of treating hypertension in PLWH and maintaining integration after the study period [38].

In regards to implementation strategies, our study does not delve deeply into the implementation strategies used by different projects to achieve integration (reported in separate publications), but common trends emerged. Capitalizing on successful strategies employed for HIV care, task shifting was broadly used across projects [42, 43]. Task shifting is a widely used method of providing counseling [44] and prescribing ART to improve access to care for HIV [45–47]. There is growing evidence of the benefits of task shifting for hypertension and CVD [48, 49]. Many projects in our study used ancillary staff, volunteer and paid lay workers to improve screening for hypertension, and trained nurses to strengthen uncomplicated hypertension management for PLWH and hypertension. This is similar to the approach used by the SEARCH study in Uganda and Kenya to facilitate integration, which used a nurse-driven triage system to tailor visits and follow-up that included appointment reminders, telephone access to clinicians, and flexible clinic hours to reduce patient level barriers [17]. To facilitate task shifting, the implementation strategy of practice facilitation, the use of coaches or experts involved in tailored training and support, was also used across projects [25, 43].

Community and Ministry of Health (MoH) engagement were commonly used strategies to guide integration model development, and to strengthen and to deliver the integrated interventions. In MAP-IT, community engagement and the use of case managers and community health extension workers was central to achieving integrated care and improving quality of care delivery [50] [51]. Though, scale up of paid and continued engagement of unpaid lay workers may also be challenging without stable financing from the government, NGOs or public-private partnerships.

Finally, the use of an iterative improvement strategy at the clinic level as used in the SCALE-SAIA-HTN project can improve clinic and system readiness to integrate services and support scale up of integration. The building blocks of the health system are continuously in flux so systems thinking powered by quality improvement can support health system resilience and continued innovation. This allows integration to continue to improve and also adjust to the always changing local context [52].

### Strengths and Limitations

Strengths of this study were the use of both questionnaires and semi-structured qualitative interviews to better understand the nuanced differences between models and the impact of environmental and logistical factors on the components of each model. Another strength was the HLB SIMPLe research community’s participation (D&I Committee) and representation from each project in designing the study and study tools. Finally, the use of the WHO Building Blocks Framework provided an appropriate and effective outline to organize and describe similarities and differences in models of HIV and hypertension integration. This framework is used widely to design, summarize and evaluate health systems strengthening initiatives. The use of this framework provides a basis for creating a uniform and reproducible model(s) of integration of HIV and hypertension care with comprehensive inclusion of the key structural components of a healthcare system [53].

A major limitation of this study was not fully evaluating leadership and governance. This building block is fundamental to maintenance and scale-up of each integration intervention. However, most projects engaged with local and/or national leadership and governance at every step of designing and getting approval for their projects. We hope to collect more data on this engagement in future work with the HLB SIMPLe Alliance.

In this study we describe the intended care models, but are not able to provide data on whether these models were implemented as intended, the degree of integration attained, or the strategies that were used to achieve and improve integration. Each project will publish this information individually as trials are completed. As individual trials are reported, additional frameworks such as the Rainbow model [54] and the Comprehensive Health Integration Framework [55] put forth recently by SAMHSA will be useful to evaluate integration quality and the degree of integration actually achieved across levels of the health system. These evaluation frameworks will also help detail the strategies used to achieve integration, explain the mechanisms by which integration happens, and describe how health systems adapt integrated care models in real-world settings [49].

## Conclusion

Our study highlights high convergence in how six implementation projects from diverse settings with differing baseline resources defined integrated care across half of the WHO Health Systems Strengthening Building Blocks. Implementation projects diverged the most for health systems financing. And, while projects were similar in describing what was being integrated, they varied in which implementation strategies were used to achieve integration. Each project adapted to the local context, a process that dictated the design of each implementation science project and integrated care model. Lessons learned from these models could help develop a common framework identifying feasible and essential building blocks for integrating NCD care into HIV programs in different (and possibly diverse) African contexts.

## Supplementary Material

Supplementary Files

This is a list of supplementary files associated with this preprint. Click to download.

- SupplementalMaterialsIntegration.docx

## Figures and Tables

**Table 1:** Integration Intervention Models by Project

Project	WHO Health System Building Blocks addressed	Evidence-based Implementation Interventions for Individuals Living with HIV and Hypertension
Integrating hypertension services into primary care HIV clinics		
InterCARE [27] (Botswana)	Health workforce Service delivery Access to basic equipment Health information systems	Four core strategies: Healthcare provider training and coaching in hypertension diagnosis and management Modification of the electronic health record to enhance hypertension and cardiovascular disease care Provision of basic equipment (such as blood pressure monitors) and hypertension guidelines Support from treatment partners (education and counseling) for hypertension
PULESA [22] (Uganda)	Health workforce Service delivery Health system financing Access to medications	Randomization into one of two core models using the following strategies in each model: Hypertension Basic: consistent access to diagnostic equipment and three evidence-based antihypertensive drugs free to hypertensive patients Hypertension-plus: Hypertension-Basic plus more intensive activities for clinic staff including training on Hypertension management, differentiated service delivery, remote patient monitoring and a performance improvement program
TASKPEN [26] (Zambia)	Health workforce Service delivery Access to medications Health information systems	Adapted version of WHO Package of Essential Non-Communicable Disease (NCD) with five core strategies: Co-location of Hypertension and HIV services (participants were able to access care for both conditions at the same place in the same visit) Training non-physician healthcare workers to accurately measure, diagnose, categorize hypertension, diabetes, and dyslipidemia. Adapting the electronic medical records used at HIV clinics to include data collection fields and decision support for NCDs. Strengthen lab point of care systems for diabetes and dyslipidemia diagnosis and monitoring. Work with the Zambia Ministry of Health to improve tracking and delivery of NCD medications to the ART clinics.
Integrating hypertension services into primary health centers		
MAP-IT [25] (Nigeria)	Health workforce	Using practice facilitation to implement a task-shifting strategy (nurses) to integrate hypertension management and treatment into primary health centers
Optimizing hypertension services in already fully integrated primary care clinics		
SCALE SAIA-HTN [24] (Mozambique)	Service delivery	An evidence-based implementation strategy that combines system engineering tools into a multi-step, facility-level package to give clinic staff and managers information on system-wide service delivery and tools to identify and prioritize areas of improvement and to test workflow modifications to achieve optimized hypertension care delivery
iHEART-SA [23] (South Africa)	Health workforce Service delivery Health information systems	Five core strategies Identifying and preparing champions (clinic care coordinators) to support and drive implementation of hypertension screening and treatment Training healthcare workers to measure, diagnose and treat hypertension Providing patient education Improving clinic flow to reduce wait times Incorporating information systems to ensure the new processes listed above are being followed

**Table 2:** Models of Integration of HIV and hypertension services as designed by each of the six projects using the WHO Health Systems Building Blocks Framework

WHO	Defined Integration Model	Integration Model Operationalized
**Building Blocks**		
Health Workforce	Same prescribing provider or clinical team for uncomplicated hypertension and HIV	All six projects had the same prescribing provider for hypertension and HIV^+#^ All six projects included nonclinical staff* as part of the clinic team to provide for routine hypertension screening and follow-up blood pressure measurement^+#^
Service Delivery	Same clinic, same visit for HIV and hypertension care	3 projects (InterCARE, PULESA, TASKPEN) added hypertension services to stand-alone HIV clinics^#^ 3 projects (iHEART-SA, SCALE-SAIA-HTN, MAP-IT) delivered HIV and hypertension together in the general primary care clinic^+^
Access to Medications	Access to essential medications with same disbursement pharmacy for HIV and hypertension medications	All six projects dispensed HIV and hypertension medications through the same pharmacy^#+^ The option to fill either HIV or hypertension medications at different local pharmacies was also available at participating study clinics (InterCARE, SCALE-SAIA-HTN and only HIV medications for iHEART-SA)^+^
Health System Financing	Using same procurement method (financing source) for both HIV and hypertension care and medications	Two (InterCARE, iHEART-SA) projects had pre-existing integrated public sector financing and procurement for both HIV and NCD medications and care delivery^+^ Four (SCALE-SAIA-HTN, MAP-IT, PULESA, TASKPEN) projects leveraged public/private partnerships for funding integrated hypertension care^+#^ Two (PULESA, TASKPEN) projects used study funding to ensure access for hypertension medications^#^
Health Information Systems	Use of the same medical record to document both HIV and hypertension clinical data and management plan	Five projects used the same medical record to document both HIV and hypertension management^+#^ (2 electronic health records (InterCARE, SCALE-SAIA-HTN), 1 paper and electronic (TASKPEN), 2 (MAP-IT, PULESA) paper records)

+ Existing infrastructure was also optimized

# New infrastructure added for the projects

* In addition to nurses in usual care for InterCARE, MAP-IT, TASKPEN, PULESA, nonclinical staff such as lay workers, CHWs and nurse assistants were included in integrated care. However, to assist in data collection, iHEART-SA included study nurses.

## Data Availability

Data available on request from the authors.

## Abbreviations

APP: advanced practice provider
ART: antiretroviral therapy
CHW: community health workers
CVD: cardiovascular disease
D&I: dissemination and implementation
HIV: human immunodeficiency virus
HLB SIMPLe Alliance: Heart, Lung, and Blood Co-morbiditieS IMplementation Models in People Living with HIV immunodeficiency virus
MO: Medical Officer
WHO: World Health Organization
PLWH: people living with HIV

## Study Acronyms:

iHEART-SA (South Africa): Integrating HIV and hEART health in South Africa
InterCARE (Botswana): Integrating Hypertension and Cardiovascular Care into Existing HIV Services Package
MAP-IT (Nigeria): Managing Hypertension Among People Living with HIV: an Integrated Model
PULESA (Uganda): Pressure Urgent Living Experience Strengthening Alliance
SCALE-SAIA-HTN (Mozambique): Scaling Out and Scaling Up the Systems Analysis and Improvement Approach to optimize the Hypertension
TASKPEN (Zambia): Task-shifted Adaptation of the WHO PEN package

## References

[1] UNAIDS. Global HIV statistics. UNAIDS 2024 epidemiological estimates. Editor: UNAIDS; 2024.

[2] Life Expectancy of Persons Receiving Combination Antiretroviral Therapy in Low-Income Countries: A Cohort Analysis From Uganda. Ann Intern Med, 2011. 155(4): pp. 209–16. 21768555 10.7326/0003-4819-155-4-201108160-00358

[3] Bor J, Increases in adult life expectancy in rural South Africa: valuing the scale-up of HIV treatment. Science. 2013;339(6122):961–5. 23430655 10.1126/science.1230413 PMC3860268

[4] Ghandakly E, Moudgil R, Holman K. Cardiovascular disease in people living with HIV: Risk assessment and management. Cleve Clin J Med. 2025;92(3):159–67. 40032300 10.3949/ccjm.92a.24055

[5] Ntsekhe M, Baker JV. Cardiovascular Disease Among Persons Living With HIV: New Insights Into Pathogenesis and Clinical Manifestations in a Global Context. Circulation. 2023;147(1):83–100. 36576956 10.1161/CIRCULATIONAHA.122.057443

[6] Xu Y, Chen X, Wang K. Global prevalence of hypertension among people living with HIV: a systematic review and meta-analysis. J Am Soc Hypertens. 2017;11(8):530–40. 28689734 10.1016/j.jash.2017.06.004

[7] Franklin SS, Wong ND. Hypertension and cardiovascular disease: contributions of the framingham heart study. Glob Heart. 2013;8(1):49–57. 25690263 10.1016/j.gheart.2012.12.004

[8] Shams P, Tackling G, Borhade MB. Hypertensive Heart Disease, in StatPearls. 2025: Treasure Island (FL). 30969622

[9] McCombe G, Integrating Care for Diabetes and Hypertension with HIV Care in Sub-Saharan Africa: A Scoping Review. Int J Integr Care. 2022;22(1):6. 10.5334/ijic.5839 PMC8815447 35136387

[10] van der Mannen JS, Lessons Learnt from HIV and Noncommunicable Disease Healthcare Integration in Sub-Saharan Africa. Glob Heart. 2024;19(1):85. 39552939 10.5334/gh.1370 PMC11568807

[11] Wroe EB, Leveraging HIV platforms to work toward comprehensive primary care in rural Malawi: the Integrated Chronic Care Clinic. Healthc (Amst). 2015;3(4):270–6. 26699356 10.1016/j.hjdsi.2015.08.002

[12] Team GCC. Integrating health services, in Technical Series: On Primary Health Care. 2018, World Health Organization.

[13] Lerberghe TEaWV. The World Health Report 2008: Primary Health Care: Now More Than Ever. Switzerland: Geneva; 2008.

[14] Walsh KF, Integrating hypertension services at an HIV clinic in Port-au-Prince, Haiti: A report from the field. J Clin Hypertens (Greenwich). 2018;20(10):1485–92. 30259642 10.1111/jch.13392 PMC6186190

[15] Muddu M, Improved hypertension control at six months using an adapted WHO HEARTS-based implementation strategy at a large urban HIV clinic in Uganda. BMC Health Serv Res. 2022;22(1):699. 35610717 10.1186/s12913-022-08045-8 PMC9131679

[16] Havlir DV, HIV Testing and Treatment with the Use of a Community Health Approach in Rural Africa. N Engl J Med. 2019;381(3):219–29. 31314966 10.1056/NEJMoa1809866 PMC6748325

[17] Hickey MD, Effect of a patient-centered hypertension delivery strategy on all-cause mortality: Secondary analysis of SEARCH, a community-randomized trial in rural Kenya and Uganda. PLoS Med. 2021;18(9):e1003803. 34543267 10.1371/journal.pmed.1003803 PMC8489716

[18] Kivuyo S, Integrated management of HIV, diabetes, and hypertension in sub-Saharan Africa (INTE-AFRICA): a pragmatic cluster-randomised, controlled trial. Lancet. 2023;402(10409):1241–50. 37805215 10.1016/S0140-6736(23)01573-8

[19] Organization WH. Integrating HIV and hypertension care for enhanced health outcome. World Health Organization: Geneva, Switzerland; 2024.

[20] Lives RtS. Integrating hypertension and HIV management: a practical differentiated service delivery toolkit. Resolve to Save Lives; 2023.

[21] Tetteh EK, Dissemination and implementation research coordination and training to improve cardiovascular health in people living with HIV in sub-Saharan Africa: the research coordinating center of the HLB-SIMPLe Alliance. Implement Sci Commun. 2024;5(1):62. 38845055 10.1186/s43058-024-00599-4 PMC11155162

[22] Longenecker CT, Implementation strategies to integrate HIV and hypertension care in Kampala and Wakiso districts, Uganda: study protocol for a stepped wedge cluster randomized trial (PULESAUganda). BMC Health Serv Res. 2025;25(1):1060. 40790739 10.1186/s12913-025-13281-9 PMC12341278

[23] Galaviz KI, Integrating hypertension detection and management in HIV care in South Africa: protocol for a stepped-wedged cluster randomized effectiveness-implementation hybrid trial. Implement Sci Commun. 2024;5(1):115. 39402688 10.1186/s43058-024-00640-6 PMC11476644

[24] Hazim CE, Scaling-up and scaling-out the Systems Analysis and Improvement Approach to optimize the hypertension diagnosis and care cascade for HIV infected individuals (SCALE SAIAHTN): a stepped-wedge cluster randomized trial. Implement Sci Commun. 2024;5(1):27. 38509605 10.1186/s43058-024-00564-1 PMC10953165

[25] Aifah AA, Study design and protocol of a stepped wedge cluster randomized trial using a practical implementation strategy as a model for hypertension-HIV integration - the MAP-IT trial. Implement Sci. 2023;18(1):14. 37165382 10.1186/s13012-023-01272-5 PMC10173657

[26] Herce ME, Evaluating a multifaceted implementation strategy and package of evidence-based interventions based on WHO PEN for people living with HIV and cardiometabolic conditions in Lusaka, Zambia: protocol for the TASKPEN hybrid effectiveness-implementation stepped wedge cluster randomized trial. Implement Sci Commun. 2024;5(1):61. 38844992 10.1186/s43058-024-00601-z PMC11155136

[27] Youssouf N, Designing an implementation science clinical trial to integrate hypertension and cardiovascular diseases care into existing HIV services package in Botswana (InterCARE). Trials. 2024;25(1):510. 39075506 10.1186/s13063-024-08333-0 PMC11285256

[28] Organization WH. Monitoring the building blocks of health systems: a handbook of indicators and their measurement strategies. World Health Organization: Geneva, Switzerland; 2010.

[29] Mokgethi O, Aligning HIV treatment and hypertension clinic visits and dispensing as a first step towards service delivery integration in South Africa. J Int AIDS Soc. 2025;28(Suppl 3):e26444. 40879611 10.1002/jia2.26444 PMC12232478

[30] Ordóñez CE, Marconi VC, Manderson L. Addressing coloniality of power to improve HIV care in South Africa and other LMIC. Front Reprod Health. 2023;5:1116813. 37064826 10.3389/frph.2023.1116813 PMC10090665

[31] Gale NK, Using the framework method for the analysis of qualitative data in multi-disciplinary health research. BMC Med Res Methodol. 2013;13(1):117. 24047204 10.1186/1471-2288-13-117 PMC3848812

[32] Palinkas LA, Mixed method designs in implementation research. Adm Policy Ment Health. 2011;38(1):44–53. 20967495 10.1007/s10488-010-0314-z PMC3025112

[33] Cameron R, Lessons from the field: Applying the good reporting of a mixed methods study (GRAMMS) framework. Electron J Bus Res Methods. 2013;11:55–66.

[34] O'Cathain A, Murphy E, Nicholl J. The quality of mixed methods studies in health services research. J Health Serv Res Policy. 2008;13(2):92–8. 10.1258/jhsrp.2007.007074 18416914

[35] Novartis. Novartis Access. 2025 June 5, 2025]; Available from: https://www.novartis.com/esg/access/creating-sustainable-business-models/novartis-access

[36] Semitala FC, Harnessing HIV clinics to deliver integrated hypertension care for People living with HIV in Uganda: A formative mixed methods study. PLOS Glob Public Health. 2025;5(6):e0004701. 40465676 10.1371/journal.pgph.0004701 PMC12136366

[37] Njuguna B, Models of integration of HIV and noncommunicable disease care in sub-Saharan Africa: lessons learned and evidence gaps. AIDS. 2018;32(Suppl 1):S33–42. 29952788 10.1097/QAD.0000000000001887 PMC6779053

[38] Njuguna B, Models of integration of HIV and noncommunicable disease care in sub-Saharan Africa: lessons learned and evidence gaps. Aids. 2018;32(1):S33–42. 29952788 10.1097/QAD.0000000000001887 PMC6779053

[39] Haldane V, Integrating cardiovascular diseases, hypertension, and diabetes with HIV services: a systematic review. AIDS Care. 2018;30(1):103–15. 28679283 10.1080/09540121.2017.1344350

[40] Matanje Mwagomba BL, Opportunities and challenges for evidence-informed HIV-noncommunicable disease integrated care policies and programs: lessons from Malawi, South Africa, Swaziland and Kenya. AIDS. 2018;32(Suppl 1):S21–32. 29952787 10.1097/QAD.0000000000001885

[41] Apeagyei AE, Financing health in sub-Saharan Africa 1990–2050: Donor dependence and expected domestic health spending. PLOS Glob Public Health. 2024;4(8):e0003433. 39196881 10.1371/journal.pgph.0003433 PMC11355530

[42] Aifah A, Nurses' perceptions on implementing a task-shifting/sharing strategy for hypertension management in patients with HIV in Nigeria: a group concept mapping study. Implement Sci Commun. 2020;1:58. 32885213 10.1186/s43058-020-00048-y PMC7427907

[43] Oladele DA, Training primary healthcare workers on a task-strengthening strategy for integrating hypertension management into HIV care in Nigeria: implementation strategies, knowledge uptake, and lessons learned. BMC Health Serv Res. 2023;23(1):673. 37344869 10.1186/s12913-023-09603-4 PMC10286327

[44] Ledikwe JH, Evaluation of a well-established task-shifting initiative: the lay counselor cadre in Botswana. PLoS ONE. 2013;8(4):e61601. 23585912 10.1371/journal.pone.0061601 PMC3621674

[45] Ivers LC, Task-shifting in HIV care: a case study of nurse-centered community-based care in Rural Haiti. PLoS ONE. 2011;6(5):e19276. 21573152 10.1371/journal.pone.0019276 PMC3089597

[46] Shumbusho F, Task shifting for scale-up of HIV care: evaluation of nurse-centered antiretroviral treatment at rural health centers in Rwanda. PLoS Med. 2009;6(10):e1000163. 19823569 10.1371/journal.pmed.1000163 PMC2752160

[47] Morris MB, Use of task-shifting to rapidly scale-up HIV treatment services: experiences from Lusaka, Zambia. BMC Health Serv Res. 2009;9:5. 19134202 10.1186/1472-6963-9-5 PMC2628658

[48] Ogedegbe G, Task shifting interventions for cardiovascular risk reduction in low-income and middle-income countries: a systematic review of randomised controlled trials. BMJ Open. 2014;4(10):e005983. 10.1136/bmjopen-2014-005983 PMC4202019 25324324

[49] Geng EH, 558Implementation Science in the Global Context: Novel Applications and Bidirectional Opportunities. Dissemination and Implementation Research in Health: Translating Science to Practice. Oxford University Press; 2023. p. 0. Brownson R.C., Colditz G.A., and Proctor E.K., Editors.

[50] Iwelunmor J, Factors influencing the integration of evidence-based task-strengthening strategies for hypertension control within HIV clinics in Nigeria. Implement Sci Commun. 2022;3(1):43. 35428342 10.1186/s43058-022-00289-z PMC9013085

[51] Mishra S, Moving forward: scaling-up the integration of an HIV and hypertension program in Akwa Ibom State, Nigeria. Glob Health Res Policy. 2024;9(1):35. 39277747 10.1186/s41256-024-00379-6 PMC11401300

[52] Gimbel S, The Systems Analysis and Improvement Approach: specifying core components of an implementation strategy to optimize care cascades in public health. Implement Sci Commun. 2023;4(1):15. 36788577 10.1186/s43058-023-00390-x PMC9926643

[53] Doherty WJ, McDaniel SH, Baird MA. Five levels of primary care/behavioral healthcare collaboration. Behav Healthc Tomorrow. 1996;5(5):25–7. 10161572

[54] Evaluation IC. The Triple Aim: Rainbow Model for Integrated Care. 2025 [cited 2025 October 15]; Available from: https://www.integratedcareevaluation.org/rainbow-model-for-integrated-care/

[55] Wellbeing NCfM. The Comprehensive Health Integration (CHI) Framework. 2025 September 29, 2025 [cited 2025 October 15]; Available from: https://www.thenationalcouncil.org/resources/thecomprehensive-health-integration-framework/

